# Reconstruction of the Genome-Scale Metabolic Model of *Saccharopolyspora erythraea* and Its Application in the Overproduction of Erythromycin

**DOI:** 10.3390/metabo12060509

**Published:** 2022-06-01

**Authors:** Feng Xu, Ju Lu, Xiang Ke, Minghao Shao, Mingzhi Huang, Ju Chu

**Affiliations:** State Key Laboratory of Bioreactor Engineering, East China University of Science and Technology, No. 130 Meilong Road, Shanghai 200237, China; fengxu@mail.ecust.edu.cn (F.X.); y45180588@mail.ecust.edu.cn (J.L.); kexianganhui@163.com (X.K.); y30200606@mail.ecust.edu.cn (M.S.)

**Keywords:** genome-scale metabolic model, *Saccharopolyspora erythraea*, iJL1426, erythromycin production, n-propanol, process optimization

## Abstract

*Saccharopolyspora erythraea* is considered to be an effective host for erythromycin. However, little is known about the regulation in terms of its metabolism. To develop an accurate model-driven strategy for the efficient production of erythromycin, a genome-scale metabolic model (iJL1426) was reconstructed for the industrial strain. The final model included 1426 genes, 1858 reactions, and 1687 metabolites. The accurate rates of the growth predictions for the 27 carbon and 31 nitrogen sources available were 92.6% and 100%, respectively. Moreover, the simulation results were consistent with the physiological observation and ^13^C metabolic flux analysis obtained from the experimental data. Furthermore, by comparing the single knockout targets with earlier published results, four genes coincided within the range of successful knockouts. Finally, iJL1426 was used to guide the optimal addition strategy of n-propanol during industrial erythromycin fermentation to demonstrate its ability. The experimental results showed that the highest erythromycin titer was 1442.8 μg/mL at an n-propanol supplementation rate of 0.05 g/L/h, which was 45.0% higher than that without n-propanol supplementation, and the erythromycin-specific synthesis rate was also increased by 30.3%. Therefore, iJL1426 will lead to a better understanding of the metabolic capabilities and, thus, is helpful in a systematic metabolic engineering approach.

## 1. Introduction

Erythromycin, a broad-spectrum macrolide antibiotic, has the advantage of acting against most Gram-positive, atypical pathogenic bacteria and is suitable for patients allergic to penicillin [1]. In addition, a series of derivatives of erythromycin, such as azithromycin, roxithromycin, and clarithromycin, have been sought for their antiparasitic, antitumor, immunosuppressive, neurotrophic, anti-inflammatory, and gastrointestinal therapeutic activities [2]. Thus, it has been occupying a crucial position in the antibiotic market. *Saccharopolyspora erythraea* (*S. erythraea*) is recognized as the primary strain of erythromycin production under the industrial-scale system. Lately, with the application of several techniques, such as random mutations, metabolic engineering modifications, and optimizations of the process conditions and media, the productivity of *S. erythraea* in the fermentation process has been significantly improved [3,4,5,6,7]. However, the industrial production of erythromycin remains inefficient compared to some other antibiotics, such as penicillin [8]. Moreover, the time spent adapting the optimal process conditions and feeding strategies to novel industrial recombinant strains is still significant, as time-consuming empirical trials are the reliable primary strategy used to achieve high-yielding processes. Although many reports have suggested the success of some rational approaches to regulation strategies, most of the mechanisms by which industrial *S. erythraea* increases its yield remain unclear. A more comprehensive understanding of cellular metabolism is urgent and necessary to enhance the erythromycin titer further.

The genome-scale metabolic model (GSMM) is a network representation of an organism’s metabolic capacity constructed from an annotated genome by using inferred or proven gene–protein–reaction (GPR) relationships, and transport reactions and estimated biomass compositions [9]. The network of reactions and metabolites in the GSMM can be represented by a stoichiometric matrix. Subsequently, the matrix is used as a core input to predict the phenotype of a specific organism subject to disturbance or its behavior in different growth environments by employing various constraint-based modeling approaches. A detailed reconstruction protocol was described in a previous report [10]. To enable an efficient and in-depth understanding of metabolic metabolism, several research groups have reconstructed a number of consensus genome-scale models (GSMMs) of *S. erythraea* [11,12]. The *S. erythraea*-specific GSMMs were built based on genomic and physiological information. Currently, there are two released GSMMs of *S. erythraea*, iZZ1342 and *S. erythraea* NRRL23338-GSMR, with the most recent one published in 2018 [12]. The iZZ1342, an upgraded version of *S. erythraea* NRRL23338-GSMR, was reconstructed based on the latest genome annotations, multi-omics databases, and annotations in the literature, which includes a more comprehensive reconstruction. Compared to the previous *S. erythraea* NRRL23338-GSMR, the iZZ1342 significantly improves the number and coverage of reactions, metabolites, and annotated genes. These models describe reaction networks that account for the conversion of all known specific substrates into biomass, erythromycin, and other metabolites. By integrating a linear-based programming framework, the GSMMs can be used to computationally determine enzyme rates under various environmental conditions. Furthermore, the GSMMs not only allow in vitro prediction of the effects of gene deletion, gene overexpression, or underexpression but can also identify metabolic targets to reduce by-product formation, and combine multi-omics data types in a computational framework [13,14].

In this study, a specific genome-scale metabolic network model, iJL1426, was developed for an industrial *S. erythraea* E3 as a support tool for interpreting experimental data to better understand cell growth and erythromycin production pathways. The iJL1426 was a more efficient and comprehensive GSMM for *S. erythraea* E3, based on the previous model iZZ1342, and supplemented and modified with knowledge gained from *S. erythraea* NRRL23338-GMR, various available databases, and multi-omics data. iJL1426 was written in level 3 of the systems biology markup language (SBML) format and is compatible with the COBRA toolbox (https://opencobra.github.io/cobratoolbox/stable/, accessed on 28 January 2021) in Matlab (MathWorks Inc., Natick, MA, USA). Then, using iJL1426 as the basis for constraint-based optimization analysis, an engineering strategy was proposed that may increase the overproduction of erythromycin due to the increased availability of the primary precursor, n-propanol. Subsequently, the optimal n-propanol feeding strategy for erythromycin overproduction in an industrial strain *S. erythraea* E3-Δ*suc*C was explored. The insights obtained in this study could serve as an efficient reference and guide further investigations of *S. erythraea* metabolism.

## 2. Results

### 2.1. Properties of the Constructed GSMM

Detailed information on the comparison results between iJL1426 and the other released GSMMs of *S. erythraea* is summarized in Table 1. The reconstructed model iJL1426 of *S. erythraea* E3 was upgraded in many aspects compared to iZZ1342. Firstly, the number of annotated genes was increased from 1342 to 1426 based on the genomic and transcriptomic data of the industrial strain E3. Secondly, the amounts of metabolic reactions were increased to 1684 after removing redundant and invalid reactions, and adding novel exchange reactions and spontaneous reactions to the model. Subsequently, the elemental and charge conservation in the metabolic reactions were corrected. Additionally, the biomass equations in iZZ1342 were corrected and upgraded by referring to the relevant literature. Finally, some vacant metabolic pathways in iZZ1342 were filled in (such as the gluconeogenesis pathway and n-propanol utilization pathway). Therefore, the results demonstrated that iJL1426 had an increase in the total number of reactions and the number of metabolites compared to iZZ1342 published in 2018, which indicated that the scale of this reconstructed model was significantly enriched. In addition, the number and proportion of annotated genes, and the number of reactions with explicit gene annotation, were boosted. The final model included 1426 genes, 1858 reactions, and 1687 metabolites. Overall, the iJL1426 was upgraded in all aspects compared to the two published versions of the model.

### 2.2. Model Validation

#### 2.2.1. Verification of Carbon and Nitrogen Source Availability

To predict the physiological state of cell growth under different scenarios, the relevant data related to phenotypic experiments were collected from previous reports and supplementary experiments. In short, 27 carbon sources and 31 nitrogen sources were validated in total. The flux balance analysis (FBA), with biomass reaction as the objective function, was employed to simulate the growth of *S. erythraea* E3 in each carbon or nitrogen source. The results demonstrated that 25 carbon sources and all nitrogen sources could support the growth of *S. erythraea* E3 based on the simulation obtained from iJL1426, which is in good agreement with the published data reported in the relevant literature and experimental data. In addition, the accuracy of iJL1426 increased by 22% and 12% compared to the previous version of the model, respectively. However, when pyruvate and acetate were applied as the only carbon sources, respectively, the simulation obtained from iZZ1342 and iJL1426 showed false negatives. This might be due to the fact that the pyruvate and acetate utilization pathway is a cellular ATP-consuming process; meanwhile, the model has no other energy source supplement. The utilization pathways of pyruvate, acetate, and several other acids were compared, in addition to the silico simulations and experimental results. The addition of these compounds might affect the extracellular pH, which, in turn, triggers changes in the metabolic mechanisms. Therefore, we hypothesize that there may be some unknown utilization pathways for pyruvate and acetate. These utilization and conversion processes for pyruvate and acetate can be addressed by subsequent experiments. For example, ^13^C-assisted isotope labeling experiments combined with genomic annotation information to find novel metabolic pathways, which can further improve the accuracy and prediction of the model. The results related to the growth of *S. erythraea* E3 on various carbon and nitrogen sources are summarized in Table 2 and Table 3.

#### 2.2.2. Verification of Physiological Metabolic Parameters

**Figure 1:**
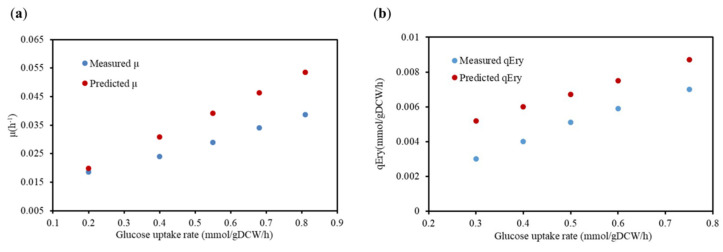
The result of the predicted and measured μ and specific erythromycin production (qEry) of *S. erythraea* E3. (**a**) The comparison results of the μ under the condition of the optimal synthetic medium; (**b**) the comparison results of the qEry under the optimal chemical-defined medium condition. Red represents the simulated results of the GSMM iJL1426, and blue represents the experimental data.

Additionally, the physiological metabolic parameters of the high-yielding strain E3 were selected as the criteria for model validation. The results illustrated in Figure 1 demonstrate that the results of the reconstructed model simulations were consistent with the in vivo experimental data. However, the simulated value was slightly higher than the experimental value, which was mainly attributed to the simulation obtained from the GSMMs under an ideal situation. Briefly, the model could avoid excess by-product generation during the simulation by optimizing the objective function, which made the carbon migration in the model more favorable to the target product. Overall, the specific growth rate of the cell and the specific erythromycin production rate exhibited the same trend, as shown in Figure 1, which essentially verified the accuracy and reliability of the model.

#### 2.2.3. ^13^C metabolic Flux Analysis Validation

**Figure 2:**
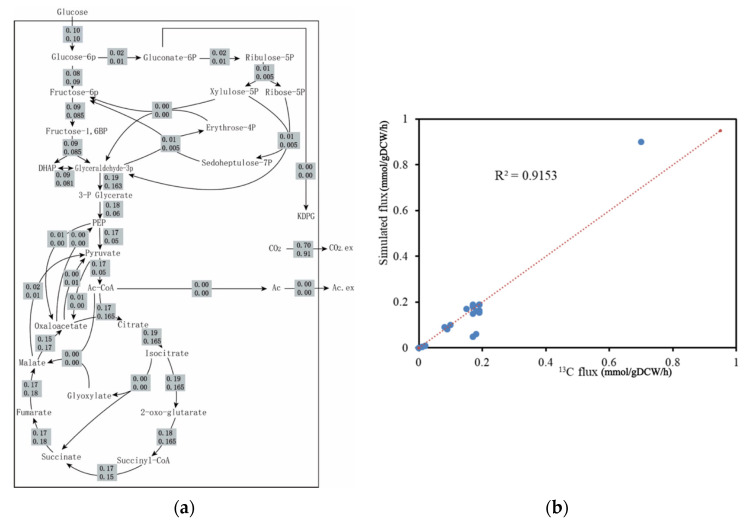
The distribution of the intracellular central metabolism flux (mmol/gDCW/h). (**a**) Metabolic flux profiles of the central metabolism of *S. erythraea*. The upper number represents the flux acquired from the ^13^C metabolic flux analysis, and the lower number represents the flux simulated from our model iJL1426; (**b**) consistent changes in fluxes can be found in both the calculated ^13^C fluxes and the FBA calculation using iJL1426.

The GSMMs play a considerable role in quantitatively predicting cellular metabolism and further exploring the enhanced properties of production strains through metabolic engineering [16]. However, there might be significant differences between the fluxes calculated in vivo and those simulated in vitro. Therefore, the fluxes obtained from metabolic flux analysis were commonly performed for comparison with the simulated results obtained from FBA to further assess the predictive accuracy of iJL1426. Here, the data of some specific metabolic pathways was obtained by performing ^13^C-labeled tracing experiments in this study, and this part of the trials was completed in our laboratory and published [7]. The flux values between the experimental data and simulation are presented in Figure 2a. The metabolic profiles obtained from the FBA simulations were consistent with those observed in the experimental measurements. Additionally, the correlation coefficient between the simulated and observed values reached 0.9153 (Figure 2b), indicating the satisfying performance of iJL1426.

#### 2.2.4. Validation of Knockout Phenotypes

Finally, the accuracy of the model was further validated by comparing the effect of target gene knockout on the erythromycin production rate. Four metabolic targets acquired from previous reports: SACE_0728 [2], SACE_0731 [17], SACE_5639 [5,18], and SACE_6669 [19], were used to simulate the single-gene knockout experiment. The comparison in Table S1 revealed that the simulations for the four selected inactivation targets were in good agreement with the previous literature. To sum up, the accuracy and predictive ability of the model were proved to be reliable and accurate by several reports and available experimental data, such as the utilization of different carbon and nitrogen sources, physiological metabolic parameters, ^13^C metabolic flux analysis results, and specific gene deletion on erythromycin profiles. Therefore, iJL1426 could be applied to perform various in silico predictions and applications.

### 2.3. Model Prediction of Essential Gene Targets In Silico for Strain Design

**Figure 3:**
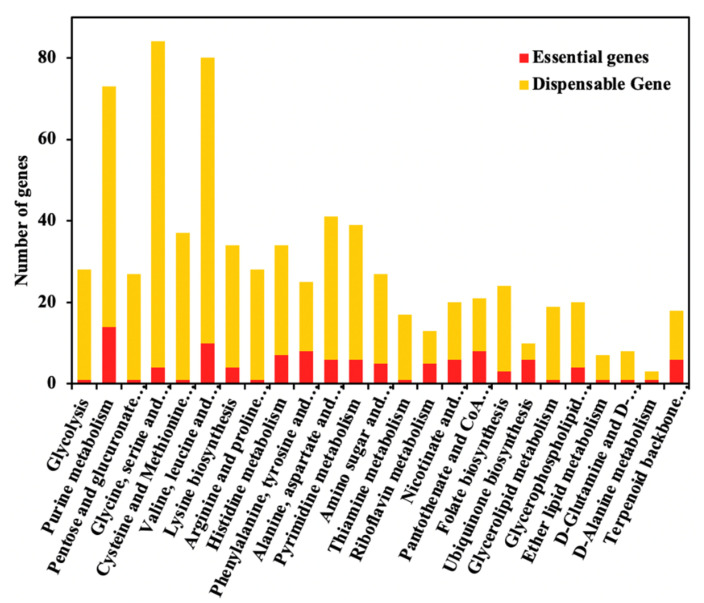
The essential genes (red) and dispensable genes (yellow) were classified in the KEGG pathway.

Compared with conventional random mutagenesis and screening, the high-quality GSMMs can combine the knowledge of genomic, kinetic, and regulatory information to locate key gene targets, thereby designing industrial strains that can enhance the production of the target metabolite [14,20]. The relationships between genes, proteins, and reactions (GPRs) established during the reconstruction of the GEM model can be applied to effectively predict genotypes by the specific desired phenotypes. In this study, the singleGeneDeletion function in COBRA Toolbox v3.0 was employed to predict essential genes and dispensable genes, which are provided in the Supplementary Materials. The prediction results demonstrated that 96 genes were regarded as essential genes and the remaining ones were dispensable ones. The number of essential genes decreased compared to the previous version of the model [12], which might be attributed to the optimal chemical-defined medium used in the simulation with more constraints. As illustrated in Figure 3, the simulations showed that the essential genes were mainly distributed in the metabolic pathways of purine and pyrimidine metabolism, energy synthesis and metabolism, amino acid synthesis, and pantothenic acid and coenzyme A synthesis.

### 2.4. Model Application of Process Optimization for n-Propanol Supplementation

#### 2.4.1. Analysis of n-Propanol Supplementation on Erythromycin Metabolism

**Figure 4:**
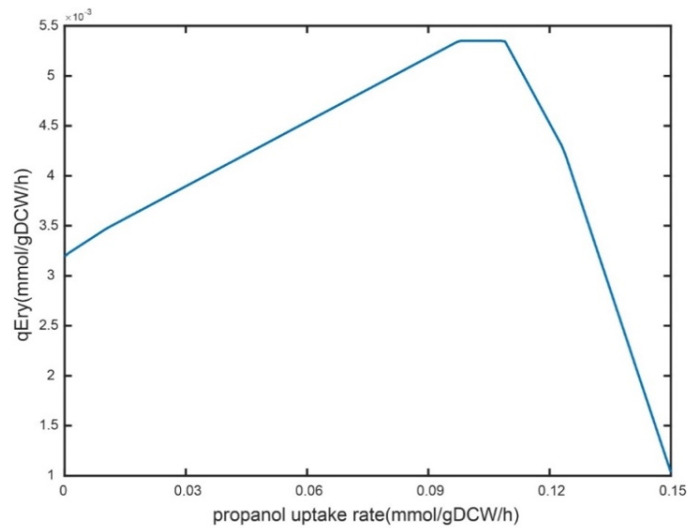
Robustness analysis of the n-propanol uptake rate to the specific erythromycin synthesis rate.

As an essential source of erythromycin precursor synthesis, n-propanol needs to be adequately supplemented in an industrial-scale fermentation system [5,8]. However, excessive residual n-propanol in the fermentation broth has some toxic effects on cellular metabolism. Here, the feeding strategy of n-propanol was optimized for an industrial strain with a rational approach. Firstly, the robustness of n-propanol supplementation was analyzed on the specific erythromycin synthesis rate using iJL1426. The results showed that the shadow price was 0.052 when the n-propanol uptake rate was 0 mmol/gDW/h, indicating that a 1 mmol/gDW/h increase in the uptake rate of n-propanol resulted in a 0.052 mmol/gDW/h enhancement in the specific erythromycin production rate (Figure 4). When the uptake rate was set to 0.04 and 0.1 mmol/gDW/h, the shadow prices were 0.022 and 0, respectively, and the shadow prices became negative as the uptake rate of n-propanol increased. Briefly, a trend that first rises and then falls in the erythromycin-specific synthesis rate was observed with the enhanced n-propanol feeding rate.

Subsequently, the intracellular metabolic flux distribution at different n-propanol uptake rates was simulated using iJL1426. The results illustrated in Figure S1 demonstrated that the majority of n-propanol entered the tricarboxylic acid (TCA) cycle from methylmalonyl CoA to succinyl CoA when the rate of n-propanol uptake was increased during the simulation, apart from the conversion into two precursors for the synthesis of erythromycin. The difference between the two simulations might be due to the fact that the set rate of glucose uptake does not produce enough ATP for the entire metabolic network. Thus, n-propanol was selected as a second carbon source to produce more ATP for the metabolic network by oxidative metabolism. Meanwhile, the carbon flux to the two precursors for erythromycin synthesis decreased, ultimately leading to a lower erythromycin-specific synthesis rate. In other words, a linear increase in erythromycin yield is not promoted by an increase in the n-propanol feeding rate.

#### 2.4.2. Cellular Physiological Parameters at Different Propanol Feeding Rates

**Figure 5:**
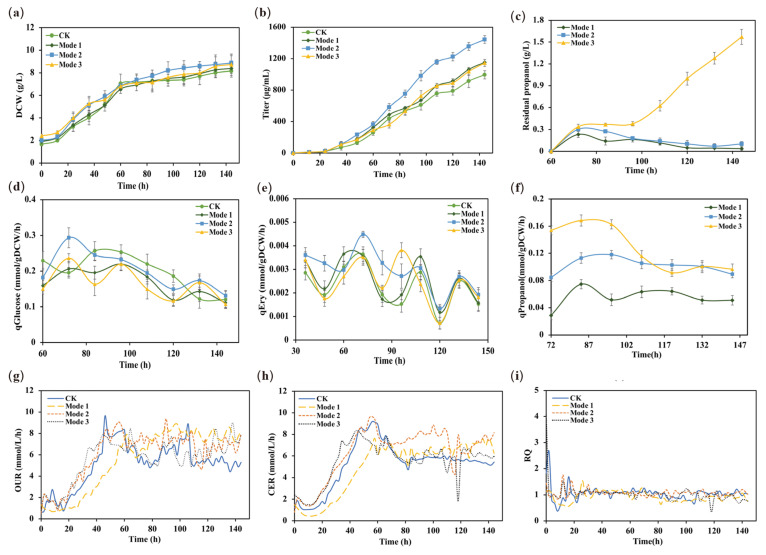
Effects of different n-propanol supplementation on metabolic parameters during the erythromycin fermentation process. (**a**) DCW (g/L); (**b**) titer of erythromycin (μg/mL); (**c**) residual propanol concentration (g/L); (**d**) specific glucose uptake rate (mmol/gDCW/h); (**e**) specific erythromycin production rate (mmol/gDCW/h); (**f**) specific propanol consumption rate (mmol/gDCW/h); (**g**) OUR (mmol/L/h); (**h**) CER (mmol/L/h); (**i**) RQ.

To further analyze the effect of the n-propanol feeding rates on the metabolism and physiology of the E3-Δ*suc*C, we compared the differences during fermentation under the three process conditions in a 5-L bioreactor (Figure 5). The cell growth in all three modes was increased during the erythromycin synthesis phase (Figure 5a), suggesting that the supplementation of n-propanol promoted the primary cellular metabolism. Moreover, the erythromycin titers were increased by 15.9%, 45.0%, and 15.2%, respectively (Figure 5b). In addition, the residual amount of n-propanol in mode 1 and mode 2 gradually decreased after 84 h, indicating that the utilization of n-propanol was higher compared to mode 3 (Figure 5c).

Subsequently, to gain insight into the effect of different n-propanol feeding rates on the metabolism of *S. erythraea* E3-Δ*suc*C, the specific glucose, n-propanol consumption, and erythromycin synthesis rates were calculated after n-propanol supplementation in the 5-L bioreactor (Figure 5d–f). The results demonstrated that the erythromycin-specific synthesis rates changed significantly after switching the n-propanol feeding rates during the fermentation process. Specifically, although the trends of the erythromycin-specific synthesis rates were the same for the four groups of fermentation modes, it was worth noting that the erythromycin synthesis rate of mode 2 was higher than the other three modes from 60–120 h of n-propanol supplementation. Then, the erythromycin synthesis rate in the four modes showed a decreasing trend at 120 h, which might be attributed to autolysis during the late stage of fermentation. From the perspective of glucose consumption, the changes showed a similar trend between the four experimental modes. However, the specific glucose consumption rate in mode 2 was slightly higher than in the other three modes after the supplementation of n-propanol in the stationary phase, indicating that the cellular physiological metabolic capacity of mode 2 was in a higher state.

Additionally, the oxygen uptake rates (OURs) were significantly higher in mode 2 and mode 3 than in mode 1 during the cell growth phase (0–40 h) (Figure 5g). The increased OUR indicated that the cellular respiration levels of mode 2 and mode 3 were elevated during the early fermentation phase. Once the cells enter secondary metabolism, the major cellular physiological activities transition to metabolic maintenance and erythromycin synthesis [21]. The carbon dioxide evolution rate (CER) of mode 2 was higher than that of the rest mode after 60 h (Figure 5h). In addition, the respiratory quotient (RQ) was used to characterize the cellular utilization capability of reduced carbon sources. Meanwhile, the levels of RQ were relatively low in all three supplementation modes (Figure 5i), indicating that the cells might have metabolized more reducing components [8,21]. The high uptake rates of n-propanol were in good agreement with the trends of CER during the rapid erythromycin formation state in model 2. Therefore, it can be concluded that the depletion of n-propanol leads to the fluctuation in CER, and more n-propanol is involved in the erythromycin synthesis pathway in model 2.

#### 2.4.3. Metabolic Flux Analysis

Although the effect of the strain on erythromycin synthesis in different modes can be analyzed by the differences in physiological metabolic rates, the detailed intracellular metabolic responses to perturbations at different n-propanol feeding rates were unclear. To analyze the effect on erythromycin synthesis in various modes of n-propanol addition in depth and provide a scientific basis for further metabolic modification and fermentation regulation, metabolic flux analysis was utilized to investigate the cellular metabolism. The rapid erythromycin synthesis phase (96–108 h) of the strain in different modes was chosen for metabolic flux analysis. In addition, the carbon recoveries were calculated in this period and ranged from 95.4–101.3%, respectively, which met the criteria for further metabolic flux analysis (Table 4).

**Figure 6:**
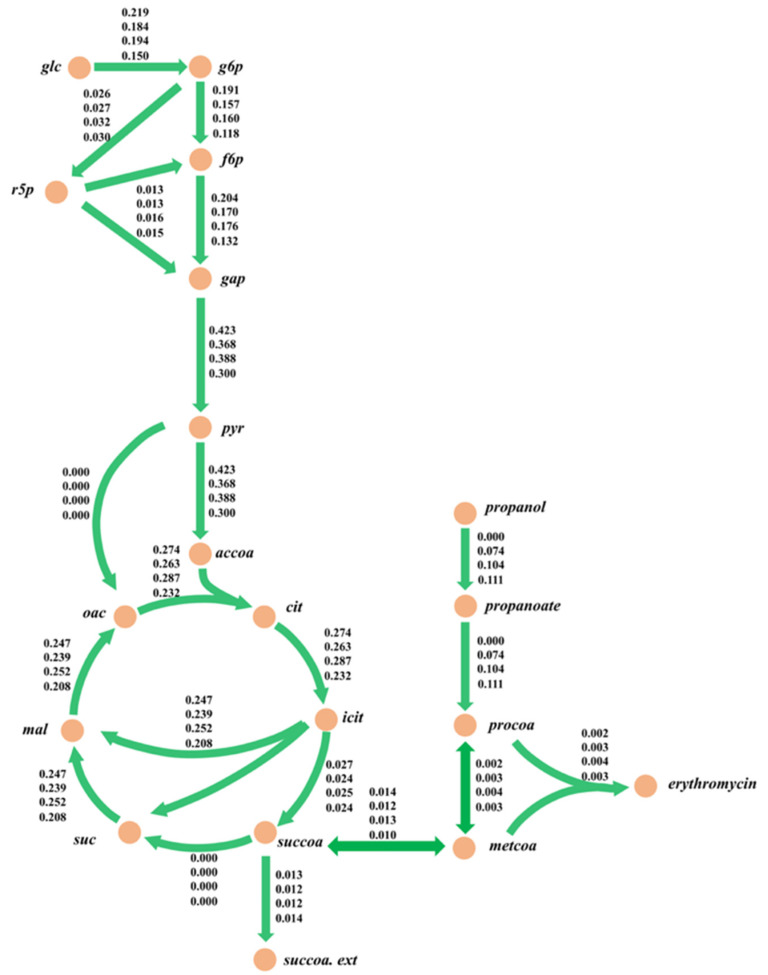
Metabolic flux analysis of each mode for the engineered strain E3-∆*suc*C during 96–108 h. The numbers near the metabolic reaction in the figure represent the metabolic flux value of the reaction; the unit is mmol/gDCW/h. The values in the figure from top to bottom are the response flux of the control group, mode 1, mode 2, and mode 3.

The results of the metabolic flux distributions are presented in Figure 6. From the perspective of substrate consumption, the specific n-propanol uptake rates gradually increased for the three feeding modes. Specifically, mode 2 had the highest uptake rate of glucose, and the rate of succinyl CoA synthesis was similar in all four modes. In terms of the central carbon metabolism, the reaction fluxes of the pentose phosphate (PP) pathway increased by 3.8%, 23.1%, and 15.4%, respectively, compared to the control group. In addition, the fluxes of the TCA cycle in mode 2 increased by 4.7% while they reduced by 4.2% and 18.1% in mode 1 and mode 3, respectively. Meanwhile, the metabolic fluxes decreased in the three modes for the glycolysis pathway. The glycolysis pathway and TCA cycle mainly provide precursors for the synthesis of the cell and ATP for cellular maintenance. Here, the cellular metabolic levels in mode 2 were higher than in the other modes, thereby causing a more significant improvement in erythromycin synthesis by increasing the energy supplement. The above results demonstrated that the experimental values of the erythromycin-specific synthesis rate were in good agreement with the simulations, which, in turn, indicated that iJL1426 had a satisfactory accuracy and predictive capability.

## 3. Discussion

With the development of the antibiotic industry, *S. erythraea* has been considered a unique and attractive strain for the industrial production of erythromycin. Here, we reconstructed and evaluated the GSMM of an industrial strain *S. erythraea* E3, named iJL1426, which contains the latest gene annotation information and can be used in constraint-based analysis. The reconstruction was based on homology with the previously reported model iZZ1342 and intensive and comprehensive manual curation of the model by complementing the model reconstruction using the automated tool modelSEED web service. We significantly expanded the metabolic scope and coverage compared to the template reconstruction of iZZ1342 and performed the reconstruction to identify the metabolic properties of *S. erythraea* E3. In addition, the connectivity of the metabolic network was dramatically improved, significantly reducing the number of blocked reactions and associated dead-end and orphan metabolites. The final version of the model represents the most comprehensive GSM reconstruction developed for this strain. Moreover, an understanding of the role of n-propanol degradation in the accumulation of coenzyme A-like precursors is critical when attempting to discern the metabolic properties of erythromycin production in *S. erythraea*. To validate the predictions of the model, the rate of n-propanol addition was optimized in this study for the pre-obtained genetically engineered strain E3-Δ*suc*C using iJL1426 at the most suitable level of the adult medium. Thus, the reconstructed model provides a valuable tool as a starting point for model-driven generation of genetic engineering and culture strategies to increase erythromycin synthesis in *S. erythraea*.

In this study, we performed labor-intensive re-annotation and comprehensive manual curation and model refinement during the model reconstruction process. Specifically, the genomic and transcriptomic data of the high-yielding strain E3 obtained from sequencing were first compared with the model strain NRRL23338 for comprehensive analysis. The incorrect gene annotation information and GPR relationship in iZZ1342 were corrected. There were 73 corrected genes, among which 51 genes without a corresponding GPR relationship in the database were deleted. After remedying the incorrect gene information, the 409 annotated genes with EC numbers that were missing after comparison with E3 were supplemented to the novel model. However, some false positives may exist. For instance, proteins involved in DNA methylation or rRNA modification also have EC numbers, but their functions are usually considered ineffective responses for metabolic network models. In total, 135 genes with valid GPR relationships were obtained and 77 GPRs relationships. Meanwhile, the curation processes were strictly followed according to the Methods and Materials sections in terms of the metabolites and reactions. Overall, the reconstructed model iJL1426 involved 1426 annotated genes, 1687 metabolites, and 1858 metabolic reactions. Compared with the previous version of the model iZZ1342, the numbers were improved by 10.5%, 2.8%, and 10.3%, respectively. Subsequently, the validation steps of the model applied data obtained from batch cultures and ^13^C-labeling experiments, making a valuable contribution to a better understanding of its physiological characteristics, as little is known about the regulation of the phenotype of *S. erythraea* E3. Specifically, the model was accessed by measuring the availability of different carbon and nitrogen sources. A remarkable enhancement in the prediction accuracy was observed at 22% and 12% compared to the iZZ1342 when using different carbon and nitrogen sources, respectively. Then, the simulation showed good agreement with the experimental data acquired from the physiological growth parameters and in vivo ^13^C metabolic flux analysis, respectively. Moreover, the predicted results showed that the inactivation of four genes increased the rate of erythromycin synthesis, which is consistent with the published studies.

The consistency, metabolic connectivity, and mass and charge balance of the reconstructions were greatly improved due to the extensive curation of the individual model components. Then, to demonstrate the application capability of the model, the iJL1426 was used to guide the optimization of the n-propanol supplementation experiments. The simulations showed that the erythromycin-specific synthesis rate was proportional to the uptake rate of n-propanol when the uptake rate of n-propanol was about 0~0.1 mmol/gDW/h. The specific synthesis rate of erythromycin was then maintained at a stable stage, and decreased as the uptake rate of n-propanol was more significant than 0.11 mmol/gDW/h. Therefore, it is vital to optimize the reasonable feeding rate of n-propanol for the erythromycin fermentation process in an industrial-scale system. As an essential precursor and key node for erythromycin synthesis, six molecules of methylmalonyl-CoA and one molecule of malonyl-CoA can synthesize one molecule of erythromycin A [22,23]. A previous study suggested that a 50% improvement in erythromycin titers could be observed by enhancing the copy number of the methylmalonic acid variant enzyme operon in wild-type strains. Therefore, it could be speculated that the synthesis rate of methylmalonyl-CoA is the rate-limiting step in erythromycin biosynthesis. Here, the flux distributions from the succinyl-CoA node to the methylmalonyl-CoA node in the four modes were 0.014, 0.012, 0.013, and 0.010 mmol/gDCW/h, respectively. Compared to the three modes of n-propanol supplementation, a higher yield of methylmalonyl-CoA, which is metabolized via the succinyl-CoA node, in mode 2 was observed. There are two ways to supply methylmalonyl-CoA to the erythromycin synthesis pathway in *S. erythraea*: namely, reversible isomerization of succinyl-CoA and carboxylation of malonyl-CoA [24,25]. In addition, the enhancement of the n-propanol feeding rate increased the rate of intracellular propionyl-CoA production by 40.5–50.0% compared to mode 1 based on the metabolic flow analysis. The metabolic flow rate to erythromycin under mode 2 increased by 33.3% compared to modes 1 and 3. It can be seen that the improvement in the erythromycin precursor concentration makes an essential contribution to the increase in erythromycin production. Moreover, it was noteworthy that the PP pathway had a significantly higher reaction flux in mode 2. Since the primary role of the PP pathway was to provide precursors for cellular growth and NADPH formation, and erythromycin was mainly synthesized during the stationary phase, in which the growth was practically stagnant, the PP pathway was used primarily to provide NADPH during erythromycin fermentation. Meanwhile, the synthesis process of erythromycin needed to consume NADPH, and the improvement in the PP pathway was beneficial as it promoted the synthesis of erythromycin [5,8,21,23]. Therefore, the results demonstrated that n-propanol at 0.05 g/L/h was the optimal feeding rate for the *S. erythraea* E3-Δ*suc*C in a 5-L bioreactor in this study, which was in good agreement with the observation obtained from the model simulation. Overall, the above results demonstrated that the iJL1426 model had satisfying accuracy and predictive ability, which provides a powerful tool and lays a solid foundation for metabolic characterization and fermentation process optimization of *S. erythraea*.

## 4. Materials and Methods

### 4.1. Microorganism, Media, and Culture Conditions

The industrial *S. erythraea* strains E3 and E3-Δ*suc*C were mainly used in this study. The culture conditions and the medium for microbial fermentation culture were the same as previously described [6,7]. Briefly, an industrial strain for erythromycin production was cultured in a 5-L bioreactor and monitored by the multi-fermenter control system Biostar (Shanghai Guoqiang Bioengineering Equipment Co., Ltd., Shanghai, China). The pH was fixed at 7.0 and controlled by 1 M NaOH solution, allowing for a variation of 0.2. The dissolved oxygen was maintained at a saturation concentration of 30%. Three feeding batches of n-propanol supplementation experiments were conducted independently in triplicate, starting with continuous feeding of n-propanol at a predetermined rate 60 h after inoculation. Specifically, the control group was supplemented with ammonium sulfate at a rate of 0.02 g/L/h until 144 h of fermentation up to 60 h, followed by mode 1, mode 2, and mode 3, all supplemented with ammonium sulfate at this flow rate. In addition, mode 1 was supplemented with n-propanol at 0.025 g/L/h; mode 2 was supplemented with n-propanol at 0.05 g/L/h; and mode 3 was supplemented with n-propanol at 0.075 g/L/h.

### 4.2. Analytical Methods

Samples were taken every 12 h from the bioreactor to determine the biomass, glucose, n-propanol consumption, and erythromycin production. Briefly, biomass was determined as the dried cell weight (DCW). Gas chromatography 7820 was used to analyze the concentration of residual n-propanol in the fermentation broth [5]. Residual glucose and erythromycin concentrations were analyzed by high-performance liquid chromatography (HPLC) as described previously [19]. Online measurements of pH, DO, OUR, and CER were achieved using dedicated custom electrodes (Mettler Toledo Co., Ltd., Zurich, Switzerland) and a process mass spectrometer (MAX300-LG, Extrel, Waltham, MA, USA), respectively.

### 4.3. iJL1426 Model Reconstruction

The reconstruction process strictly followed the protocol recommended by Thiele and Palsson [10]. In this study, the iJL1426 was improved and upgraded in four aspects compared to the previous version of the model. Firstly, the reactions were re-annotated with the latest genomic annotation information (JABNNH000000000) specified for the high-yielding strain *S. erythraea* E3 to ensure the model was more suitable for industrial production. Then, the newest transcriptomics data (GSE134767) measured for *S. erythraea* E3 was used to perform preliminary manual curation and refinement of the model, including unifying the metabolite names and removing invalid and duplicate metabolic reactions. Subsequently, the charge and mass conservation of metabolites in the iZZ1342 model were checked in combination with several databases. Moreover, the biomass synthesis reactions were modified by referring to the relevant literature [7].

#### 4.3.1. Draft Model Reconstruction

The genome-scale metabolic network reconstruction for *S. erythraea* E3 was based on the latest published genome annotation and carried out using the ModelSEED (https://modelseed.org/, accessed on 16 January 2021) [26], which was a semi-automatic and open-source application based on the Rapid Annotation of microbial genomes using Subsystems Technology (RAST) for reconstructing, exploring, comparing, and analyzing metabolic models [27]. By uploading the latest gene annotation results to the ModelSEED program, the system returns an automatically constructed draft model.

#### 4.3.2. Gap-Filling

Subsequently, the model was then imported into MATLAB for manual curation using the COBRA toolbox [28]. Gap-filling was performed to yield a reliable and functional model using the fastGapFill function. Since some metabolites were not fully utilized, the accumulation of these metabolites can block the pathways and make the model inoperable. Therefore, it was necessary to provide the corresponding exchange reactions. In addition, a class of reactions existed in cells that did not require enzyme catalysis but could proceed spontaneously, named spontaneous reactions. After referring to the literature and databases, some of these reactions were missing in the iZZ1342 model and were manually supplemented in the reconstructed model. These reactions were not explicitly annotated with genes; thus, the corresponding GPR relationships might be missing but were essential for the integrity of the model. Some spontaneous reactions were accordingly incorporated based on the gaps presented in the model, which avoided the generation of new gaps due to the addition of spontaneous reactions, and then referred to multiple databases to annotate the added spontaneous reactions.

#### 4.3.3. Curation of Directionality and Reversibility

Since a wrong direction may lead to a severe decrease in the accuracy of the model, we then corrected the directionality and reversibility of the metabolic reactions after constructing a draft model. Here, the eQuilibrator was employed to calculate changes in the Gibbs free energy under standard conditions to infer the directionality of the reactions [29]. Specifically, we assumed that most reactions were reversible unless the eQuilibrator predicted a significant change in the Gibbs free energy (>30 kJ/mol) under standard conditions (25 °C, 1 bar), pH 7.0, and 1mM concentration of reactants [30,31]. Meanwhile, an alternative approach was to combine the thermodynamic information with network topology and heuristic rules to assign the directionality of the reaction [32]. Additionally, various practical and feasible rules were applied to check or correct the directionality of the reactions in the model [33].

#### 4.3.4. Manual Refinement

A large number of invalid and redundant reactions in the draft model were checked and evaluated. These reactions were divided into three prominent cases: first, the bias of the original gene annotation information led to the deletion of GPRs with inappropriate relationships; second, the correction of cofactors in the reactions led to the omission of redundant reactions; third, the crossover phenomenon of two different metabolic pathways led to the deletion, for instance, the same reaction appears in both metabolic pathways. The presence of these reactions means the model did not work correctly or fell into an infinite loop, so it was identified and deleted in time during the model refinement stage.

Additionally, the elemental and charge conservation in the metabolic reaction was checked by the relevant scripts in MATLAB, which were available on the GitHub repository (https://github.com/FengxuSysbio/Sery-GEM, accessed on 16 May 2022). If the element or charge in the reaction is not conserved, two cases should be considered: first, the charge or molecular formula of the metabolite is wrong, in which case multiple databases should be searched for confirmation, and the proton [H] should be added on both sides of the reaction to ensure balance; second, the stoichiometric coefficients of the metabolic reaction itself are not balanced. In such cases, it may be necessary to add protons [H] or water to the reaction and then perform stoichiometric coefficients’ equilibrium to satisfy the reaction balance. Therefore, each element and charge should be balanced on both sides of the reaction.

#### 4.3.5. Biomass Reactions

Defining the biomass composition is essential for optimal metabolic network performance of the target organism. The biomass reaction in iZZ1342 was mainly referred to and taken from iMK1208 and iJO1366 [34,35]. However, the differences in the biomass composition between various strains could decrease the accuracy of the model. Here, the components of the cellular biomass (DNA, RNA, carbohydrates, proteins, and lipids) were obtained from the published data by our group [7]. The biomass synthesis reactions are presented in the supporting information.

### 4.4. In Silico Computation Using Flux Balance Analysis

The constraint-based FBA was performed using the COBRA toolbox and Gurobi^®^ Optimizer version 9.1.1 as an optimization solver for linear programming, in which the stoichiometric matrix inside the metabolic network was the source of constraints. The optimal solution of the system in the space of feasible flux solutions was computed and obtained using linear programming algorithms. The biological phenotype of the optimal solution in this paper was the maximization of the cell growth or specific product synthesis rate. This problem was represented using the matrix notation and is stated as Equations (1)–(3) [36,37]:

(1) $$maximize:\;c^{T}\cdot v$$

(2) Constraints:S·v=0

(3) $$v_{min}\leq v\leq v_{max}$$

where *S* refers to a stoichiometric matrix representing the matrix composed of the stoichiometric coefficients of metabolites in the metabolic reactions; *v* represents the vector composed of the metabolic reactions’ fluxes; *v_min_* and *v_max_* are the minimum and maximum constraints, which are defined as the maximum enzymatic reaction rate and the reversibility of metabolic reactions, respectively; and *c^T^* refers to a weight vector, indicating the contribution of each reaction (such as the biomass reaction at the simulated maximum growth rate) to the objective function. Glucose was chosen as the sole carbon source in the simulations. Other extracellular metabolites, such as H_2_O, O_2_, CO_2_, PO_4_^3−^, NH^4+^, and SO_4_^2−^, were set to be transported freely across the membrane intracellularly and extracellularly. All simulations were performed on the MATLAB 2019b platform.

### 4.5. Model Validation

The results of the model simulation and several experiments were utilized to fully assess the predictive accuracy of iJL1426. The physiological data and ^13^C metabolic flux analysis results were obtained from previous publications and experiments in this study [12,15]. Briefly, the specific substrate was considered to support growth if the predicted solution was significantly higher than zero. In addition, the simulated value of 10^−5^ h^−1^ was used to determine whether the cell could grow or not.

Moreover, the accuracy and reliability of the model were visually assessed by comparing the experimental values of the cellular-specific growth rate and the erythromycin-specific synthesis rate with the model simulation data. When simulating the specific growth rate, it was necessary to ensure that the substrates and other parameters required for the growth were consistent with those under experimental conditions, such as water, hydrogen ion, sulfate ion, ammonium ion, phosphate ion, specific oxygen consumption rate, and specific carbon dioxide synthesis rate. When simulating the specific synthesis rate of erythromycin, in addition to constraining the required essential nutrients and specific oxygen consumption rate and specific carbon dioxide synthesis rate, it was also vital to set the specific growth rate to be consistent with the experimental conditions; that is, to simulate the specific synthesis rate of erythromycin at the same specific growth rate.

### 4.6. Model Simulation and Analysis

The essential and dispensable genes were predicted using the *singleGeneDeletion* function based on the COBRA toolbox [12,28]. The flux of the reaction containing the gene of interest was set to zero during the essential gene analysis according to the matrix of GPR and the definition of the Boolean rule. In contrast, the bounds of the other reactions were kept constant. Genes were classified into two groups (essential genes and dispensable genes) based on the magnitude of the specific growth rate calculated when knocking out a gene. An optimized chemically defined media was used to analyze these genes [6].

## 5. Conclusions

In this study, we developed and constructed a high-quality GSM of the *S. erythraea* E3, called iJL1426. The novel model contains 1426 genes, 1687 metabolites, and 1858 reactions. The model provides the most comprehensive knowledge base on the biochemistry of *S. erythraea* to date, with a particular emphasis on n-propanol metabolism. By manually organizing the model on a large scale, we greatly improved the coverage and scope of metabolism based on previously published GSMMs. With the reformulated biomass composition, the model was able to qualitatively reproduce growth phenotypes on multiple experimentally tested nutrient sources and accurately predict and identify some engineering targets for improved erythromycin productivity. In addition, the effect of n-propanol on the erythromycin-specific synthesis rate was analyzed under the guidance of the reconstructed GSMM to optimize the existing n-propanol supplementation process for the genetically engineered strain E3-Δ*suc*C. We strongly believe that the reconstructed model will form a solid framework to explore and understand the metabolic properties of *S. erythraea* E3 and help generate metabolic engineering and culture strategies in vitro to improve erythromycin productivity. Furthermore, the model contains a quality control and assurance scaffold for future reconstruction of strain-specific *S. erythraea* GSMs important for biotechnology and industrial applications.

## Figures and Tables

**Table 1:** Comparison of the main characteristics of *S. erythraea*.

Characteristics	iJL1426	iZZ1342 [12]	NRRL23338-GEMR [11]
Genome size	8.2Mb	8.2 Mb	8.2 Mb
Total genes	7714	7233	7233
Genes assigned	1426	1342	1272
effective genes	1426	1291	1272
Annotation coverage (%)	18.5%	17.9%	17.5%
Total reactions	1858	1684	3985
Unique reactions	1858	1611	1482
Metabolic reactions	1632	1525	3872
Transport and exchange reactions	225	133	113
Metabolites	1687	1614	1546
GPR associations	1492	1441	-
Reactions with genes assigned	1492	1441	1223
Reactions without genes assigned	366	243	2762

**Table 2:** Prediction of the ability of *S. erythraea* E3 to utilize different carbon sources (+ represents growth and – represents non-growth).

Carbon Source	Observed in Experiment	Predicted in Model	Reference
D-Glucose	+	+	[15]
sucrose	+	+	[15]
D-Xylose	+	+	[15]
Mannose	+	+	[12]
Mannitol	+	+	[12]
L-Rhamnose	+	+	[12]
L-Arabinose	+	+	[15]
D-Mannose	+	+	[12]
D-Fructose	+	+	[15]
Raffinose	+	+	[12]
D-Galactose	+	+	[15]
inost	+	+	[12]
Melibiose	+	+	[12]
D-Ribose	+	+	[15]
alpha,alpha-Trehalose	+	+	[15]
Maltose	+	+	[15]
β-Lactose	+	+	[15]
α-Lactose	+	+	[15]
Pyruvate	+	−	[12]
2-Oxoglutarate	−	−	[12]
Succinate	−	−	[12]
Fumarate	−	−	[12]
Acetate	+	−	[12]
Propanoate	+	+	[12]
Citrate	+	+	[12]
(S)-Malate	+	+	[12]
(S)-Lactate	−	−	[12]

**Table 3:** Prediction of the ability of *S. erythraea* E3 to utilize different nitrogen sources (+ represents growth and – represents non-growth).

Nitrogen Source	Observed in Experiment	Predicted in Model	Reference
L-Valine	+	+	[12]
L-Threonine	+	+	[12]
L-Isoleucine	+	+	[12]
L-Leucine	+	+	[12]
L-Methionine	+	+	[12]
L-Aspartate	+	+	[12]
L-Glutamine	+	+	[12]
L-Phenylalanine	+	+	[12]
L-Glutamate	+	+	[12]
L-Serine	+	+	[12]
L-Proline	+	+	[12]
Glycine	+	+	[12]
L-Lysine	−	−	[12]
L-Histidine	+	+	[12]
L-Cysteine	+	+	[12]
L-Asparagine	+	+	[12]
L-Alanine	+	+	[12]
L-Arginine	+	+	[12]
L-Tyrosine	−	−	[12]
L-Tryptophan	+	+	[12]
Urea	+	+	[12]
4-Aminobutanoate	+	+	[12]
Xanthine	−	−	[12]
Hypoxanthine	−	−	[12]
Ammonium chloride	+	+	[12]
Ammonium nitrate	+	+	[15]
Ammonium acetate	+	+	[15]
Ammonium oxalate	+	+	[15]
Ammonium carbonate	+	+	[15]
Ammonium sulfate	+	+	[12]
Ammonium dihydrogen phosphate	+	+	[15]

**Table 4:** Variation rate and carbon recovery rate of each group of engineered strains E3-∆sucC during 96–108 h.

	CK	Mode 1	Mode 2	Mode 3
qGlucose (mmol/gDCW/h)	0.219 ± 0.003	0.184 ± 0.002	0.194 ± 0.002	0.150 ± 0.001
qpropanol (mmol/gDCW/h)	0	0.073 ± 0.003	0.105 ± 0.005	0.115 ± 0.005
qCO_2_ (mmol/gDCW/h)	0.826 ± 0.004	0.837 ± 0.003	0.968 ± 0.005	0.701 ± 0.005
qEry (mmol/gDCW/h)	0.002 ± 0.001	0.003 ± 0.001	0.003 ± 0.001	0.003 ± 0.001
qsuc-coA (mmol/gDCW/h)	0.013 ± 0.001	0.012 ± 0.001	0.012 ± 0.002	0.014 ± 0.001
μ(h-1)	0.001 ± 0.001	0.001 ± 0.001	0.002 ± 0.001	0.001 ± 0.001
Carbon recoveries (%) *	95.5	96.6	97.4	95.8

* Carbon recoveries = [(qCO_2_ ∗ 1 + qEry ∗ 37 + qsuc-coA ∗ 25 + μ ∗ 1000/32.75)/(qGlucose ∗ 6 + qpropanol ∗ 3)] ∗ 100%.

## Data Availability

The model files, reconstruction pipeline, and relevant data can be found at https://github.com/FengxuSysbio/Sery-GEM (accessed on 16 May 2022).

## Supplementary Materials

The following supporting information can be downloaded at: https://www.mdpi.com/article/10.3390/metabo12060509/s1, Figure S1: Visualization diagram of n-propanol utilization pathway in metabolic network model iJL1426. The red line in (a) is the utilization pathway of low-concentration n-propanol feeding rate and (b) the utilization pathway of high-concentration n-propanol feeding rate; Table S1: The protein and biomass reaction for the *S. erythraea* E3; Table S2. Information on 96 essential genes was obtained from model simulations.
