# Supplementary material for: Reconstruction of the Genome-Scale Metabolic Model of Saccharopolyspora erythraea and Its Application in the Overproduction of Erythromycin
Source: Metabolites. 2022 Jun 1;12(6):509. doi: 10.3390/metabo12060509 (PMC9228414; doi:10.3390/metabo12060509)
Supplement: Supplementary file 1 [file metabolites-12-00509-s001.zip › metabolites-1751783-supplementary.pdf]

Reconstruction of the genome-scale metabolic model of *Saccharopolyspora erythraea* and its application in the overproduction of erythromycin

Feng Xu[1], Ju Lu[1], Xiang Ke[1], Minghao Shao[1], Mingzhi Huang[1,*], Ju Chu[1]

[1] State Key Laboratory of Bioreactor Engineering, East China University of Science and Technology,

130 Meilong Road, Shanghai, 200237, People's Republic of China

* Corresponding author: Mingzhi Huang

E-mail address: huangmz@ecust.edu.cn

Supplemental information

Fig. S1. Visualization diagram of n-propanol utilization pathway in metabolic network model iJL1426. The red line in (a) is the utilization pathway of low-concentration n-propanol feeding rate and (b) the utilization pathway of high-concentration n-propanol feeding rate.

Table S1. The protein and biomass reaction for the *S. erythraea* E3.

Table S2. Information on 96 essential genes obtained from model simulations.

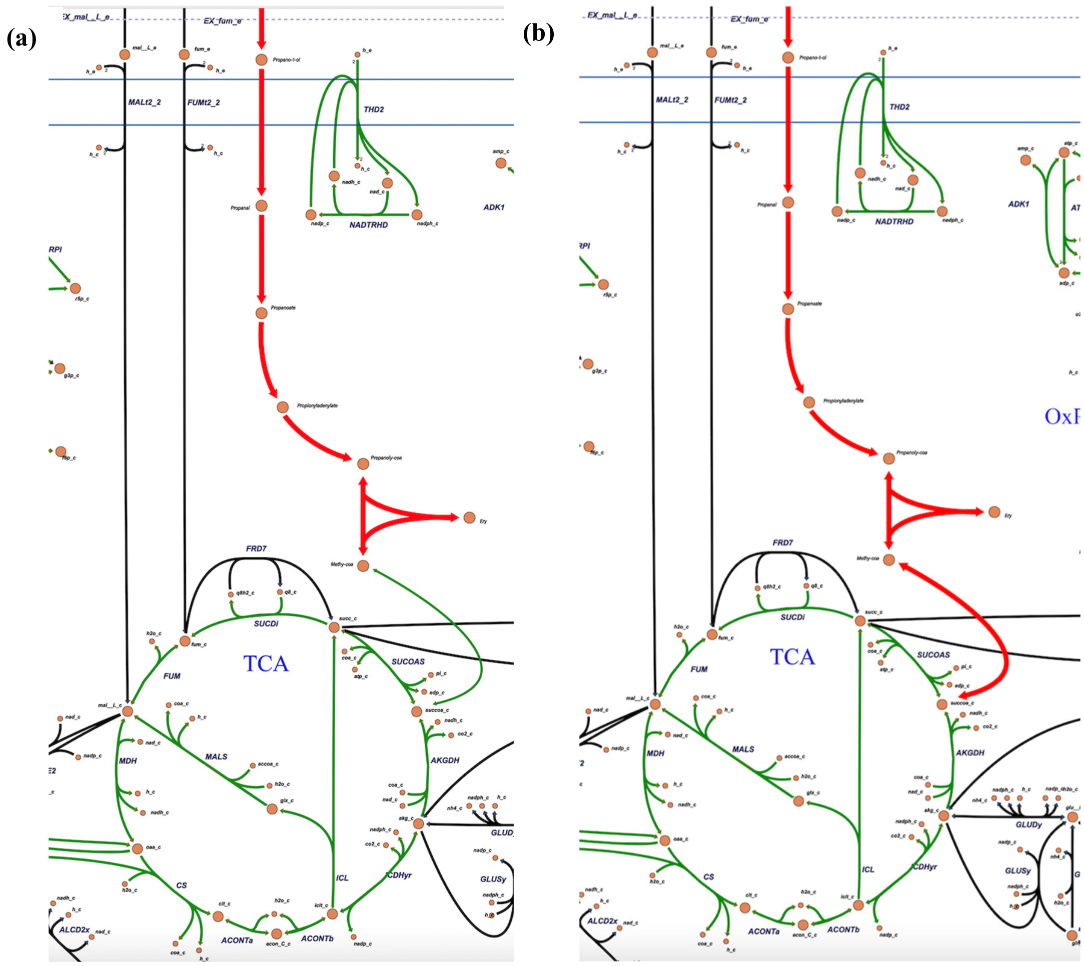

(a)
EX_mal__L_e
EX_fum_e
MALt2_2
FUMt2_2
THD2
NADTRHD
ADK1
Propanal
Propanoate
Propionyladenylate
Propanoly-coa
Methy-coa
Ery
FRD7
SUCDi
FUM
TCA
SUCOAS
MDH
MALS
AKGDH
CS
ICL
ICDHyr
ACONTa
ACONTb
ALCD2x
GLUSy
(b)
EX_mal__L_e
EX_fum_e
MALt2_2
FUMt2_2
THD2
NADTRHD
ADK1
Propanal
Propanoate
Propionyladenylate
Propanoly-coa
Methy-coa
Ery
FRD7
SUCDi
FUM
TCA
SUCOAS
MDH
MALS
AKGDH
GLUDy
GLUSy
CS
ICL
ICDHyr
ACONTa
ACONTb
ALCD2x

(Figure S1)

Table S1. The protein and biomass reaction for the *S. erythraea* E3.

	Reactions
Protein	1.068 L-Alanine[c] + 0.237 L-Asparagine[c] + 0.025 L-Arginine[c] +0.519 L-Glutamate[c] + 0.081 L-Glutamine[c] + 0.676 Glycine[c] + 0.28 L-Serine[c] + 0.129 L-Valine[c] + 0.409 L-Isoleucine[c] + 0.251 L-Threonine[c] + 0.081 L-Lysine[c] + 0.677 L-Aspartate[c] + 0.131 L-Cysteine[c] + 0.201 L-Methionine[c] + 0.071 L-Leucine[c] + 0.103 L-Phenylalanine[c] + 0.213 L-Tryptophan[c] + 0.044 L-Tyrosine[c] + 0.381 L-Proline[c] + 0.075 L-Histidine[c] + 40 ATP[c] => Protein + 40 ADP[c] + 40 Orthophosphate[c]
Biomass	0.412 Protein[c] + 0.036 DNA[c] + 0.167 RNA[c] + 0.027 Phospholipids[c] + 0.064 Carbohydrate[c] + 0.125 Peptidoglycan[c] + 0.018 TAG[c] + 0.03 Cofactor[c] + 0.031 Teich[c] + 64 ATP[c] => biomass[c] + 64 ADP[c] + 64 Orthophosphate[c]

Table S2. Information on 96 essential genes obtained from model simulations.

ORF	Reaction	Gene function	Pathway
SACE_0231	Prephenate[c] + NAD[c] <=> 34hpp[c] + CO2[c] + NADH[c] + H[c]	prephenate dehydrogenase	Phenylalanine, tyrosine and tryptophan biosynthesis
SACE_0282	ATP[c] + L-Aspartate[c] => ADP[c] + 4-Phospho-L-aspartate[c]	aspartate kinase	Glycine, serine and threonine metabolism
SACE_0283	4-Phospho-L-aspartate[c] + NADPH[c] + H[c] => aspsa[c] + Orthophosphate[c] + NADP[c]	aspartate-semialdehyde dehydrogenase, USG-1 related	Glycine, serine and threonine metabolism
SACE_0399	ahd[c] => Glycolaldehyde[c] + 2ahhmp[c]	dihydroneopterin aldolase	Folate biosynthesis
SACE_0439	2me4p[c] + CTP[c] <=> 4c2me[c] + Diphosphate[c]	4-diphosphocytidyl-2C-methyl-D-erythritol synthase	Terpenoid backbone biosynthesis
SACE_0440	2-Phospho-4c2me[c] <=> 2mecdp[c] + CMP[c]	2mecdp synthase	Terpenoid backbone biosynthesis
SACE_0541	(1R,6R)-6-Hydroxy-2-succinylcyclohexa-2,4-diene-1-carboxylate[c] => 2-Succinylbenzoate[c] + H2O[c]	O-succinylbenzoate synthase	Ubiquinone biosynthesis
SACE_0591	Thioredoxin[c] + GDP[c] => dGDP[c] + trdds[c] + H2O[c]	SACE_0591ribonucleotide-diphosphate reductase subunit beta	Purine metabolism
SACE_0604	Pyruvate[c] + 2-Oxobutanoate[c] => (S)-2-Aceto-2-hydroxybutanoate[c] + CO2[c]	acetolactate synthase large subunit	Valine, leucine and isoleucine biosynthesis
SACE_0673	1-Acylglycerol[c] + H2O[c] => Glycerol[c] + fa[c]	SACE_0673lipase	Glycerolipid metabolism
SACE_0774	UTP[c] + g1p[c] => Diphosphate[c] + udpg[c]	UTP--glucose-1-phosphate uridylyltransferase	Pentose and glucuronate interconversions
SACE_0807	4c2me[c] + ATP[c] <=> 2-Phospho-4c2me[c] + ADP[c]	4-diphosphocytidyl-2-C-methyl-D-erythritol kinase	Terpenoid backbone biosynthesis
SACE_0817	Acetyl-CoA[c] + gam1p_A[c] => CoA[c] + acgam1p[c]	putative UDP-N-acetylglucosamine pyrophosphorylase	Amino sugar and nucleotide sugar metabolism

SACE_1013	Succinyl-CoA[c] + thdp[c] + H2O[c] => CoA[c] + sl2a6o[c]	2,3,4,5-tetrahydropyridine-2,6-dicarboxylate N-succinyltransferase	Lysine biosynthesis
SACE_1018	sl26da[c] + H2O[c] => Succinate[c] + 26dap_LL[c]	dipeptidase	Lysine biosynthesis
SACE_1282	Thioredoxin[c] + CDP[c] => dCDP[c] + trdds[c] + H2O[c]	ribonucleoside-diphosphate reductase, beta subunit	Purine metabolism
SACE_1283	Thioredoxin[c] + UDP[c] => dUDP[c] + trdds[c] + H2O[c]	ribonucleoside-diphosphate reductase, alpha subunit	Pyrimidine metabolism
SACE_1387	ru5p_D[c] <=> r5p_D[c]	ribose 5-phosphate isomerase RpiB	Pentose phosphate pathway
SACE_1401	ATP[c] + CDP[c] <=> ADP[c] + CTP[c]	nucleoside diphosphate kinase	Purine metabolism
SACE_1428	ATP[c] + nicrnt[c] <=> Diphosphate[c] + Deamino-NAD[c]	putative nicotinate-nucleotide adenylyltransferase	Nicotinate and nicotinamide metabolism
SACE_1610	ATP[c] + Deamino-NAD[c] + L-Glutamine[c] + H2O[c] => AMP[c] + Diphosphate[c] + NAD[c] + L-Glutamate[c]	glutamine-dependent NAD(+) synthetase	Nicotinate and nicotinamide metabolism
SACE_1681	Anthranilate[c] + prpp[c] <=> pran[c] + Diphosphate[c]	anthranilate phosphoribosyltransferase	Phenylalanine, tyrosine and tryptophan biosynthesis
SACE_1753	26dap_LL[c] <=> 26dap_M[c]	diaminopimelate epimerase	Lysine biosynthesis
SACE_1764	Thioredoxin[c] + UDP[c] => dUDP[c] + trdds[c] + H2O[c]; Thioredoxin[c] + ADP[c] => dADP[c] + trdds[c] + H2O[c]	ribonucleoside diphosphate reductase,B12-dependent	Purine metabolism
SACE_1802	2 Orthophosphate[c] + D-Glucose[c] => Orthophosphate[c] + g6p[c]	polyphosphate glucokinase	Glycolysis
SACE_1848	N-Acetyl-D-mannosamine[c] + Orthophosphate[c] => acmanap[c] + H2O[c]	hydrolase or phosphatase	Amino sugar and nucleotide sugar metabolism
SACE_2065	3psme[c] => Chorismate[c] + Orthophosphate[c]	chorismate synthase	Phenylalanine, tyrosine and tryptophan biosynthesis
SACE_2066	ATP[c] + Shikimate[c] => ADP[c] + skm3p[c]	shikimate kinase I	Phenylalanine, tyrosine and tryptophan biosynthesis

SACE_2079	cbp[c] + L-Aspartate[c] => Orthophosphate[c] + cbasp[c] + H[c]	aspartate carbamoyltransferase catalytic chain	Alanine, aspartate and glutamate metabolism
SACE_2080	cbasp[c] => (S)-Dihydroorotate[c] + H2O[c]	dihydroorotase	Pyrimidine metabolism
SACE_2082	2 ATP[c] + L-Glutamine[c] + HCO3[c] + H2O[c] => 2 ADP[c] + Orthophosphate[c] + L-Glutamate[c] + cbp[c]	carbamoyl-phosphate synthase small subunit	Alanine, aspartate and glutamate metabolism
SACE_2083	2 ATP[c] + L-Glutamine[c] + HCO3[c] + H2O[c] => 2 ADP[c] + Orthophosphate[c] + L-Glutamate[c] + cbp[c]	carbamoyl-phosphate synthase small subunit	Alanine, aspartate and glutamate metabolism
SACE_2084	orot5p[c] => UMP[c] + CO2[c]	orotidine-5prime-phosphate decarboxylase	Pyrimidine metabolism
SACE_2100	ATP[c] + dGMP[c] <=> ADP[c] + dGDP[c]	guanylate kinase	Purine metabolism
SACE_2122	2 dmlz[c] => Riboflavin[c] + 5-Amino-6-(1-D-ribitylamino)uracil[c] + H[c]	riboflavin synthase subunit alpha	Riboflavin metabolism
SACE_2123	ru5p_D[c] => db4p[c] + Formate[c]	bifunctional db4p synthase/GTP cyclohydrolase II protein	Riboflavin metabolism
SACE_2124	5-Amino-6-(1-D-ribitylamino)uracil[c] + db4p[c] => dmlz[c] + 2 H2O[c] + Orthophosphate[c]	riboflavin synthase beta chain	Riboflavin metabolism
SACE_2136	(S)-Dihydroorotate[c] + O2[c] <=> Orotate[c] + H2O2[c]	dihydroorotate dehydrogenase	Pyrimidine metabolism
SACE_2237	1-(5-Phospho-D-ribosyl)-ATP[c] + H2O[c] => Phosphoribosyl-AMP[c] + Diphosphate[c]	phosphoribosyl-ATP pyrophosphatase	Histidine metabolism
SACE_2238	ATP[c] + prpp[c] <=> 1-(5-Phospho-D-ribosyl)-ATP[c] + Diphosphate[c]	ATP phosphoribosyltransferase	Histidine metabolism
SACE_3465	Pyruvate[c] + 2-Oxobutanoate[c] => (S)-2-Aceto-2-hydroxybutanoate[c] + CO2[c]	acetolactate synthase large subunit	Valine, leucine and isoleucine biosynthesis
SACE_3848	Diphosphate[c] + Propionyladenylate[c] <=> ATP[c] + Propanoate[c]	acetyl-CoA synthetase	Propanoate metabolism
SACE_3898	5mthf[c] + L-Homocysteine[c] <=> Tetrahydrofolate[c] + L-Methionine[c]	putative 5mthf:homocysteine S-methyltransferase	Cysteine and Methionine metabolism

SACE_3908	Pyruvate[c] + 2-Oxobutanoate[c] => (S)-2-Aceto-2-hydroxybutanoate[c] + CO2[c]	acetolactate synthase large subunit	Valine, leucine and isoleucine biosynthesis
SACE_4565	Pyruvate[c] + 2-Oxobutanoate[c] => (S)-2-Aceto-2-hydroxybutanoate[c] + CO2[c]	acetolactate synthase large subunit	Valine, leucine and isoleucine biosynthesis
SACE_5234	ADP[c] + CDP[c] <=> ATP[c] + CMP[c]	cytidylate kinase	Pyrimidine metabolism
SACE_5244	ATP[c] + UTP[c] + NH3[c] => ADP[c] + Orthophosphate[c] + CTP[c]	CTP synthase	Pyrimidine metabolism
SACE_5249	ATP[c] + NAD[c] => ADP[c] + NADP[c]	SACE_5249putative inorganic polyphosphate/ATP-NAD kinase	Nicotinate and nicotinamide metabolism
SACE_5632	Pyruvate[c] + 2-Oxobutanoate[c] => (S)-2-Aceto-2-hydroxybutanoate[c] + CO2[c]	acetolactate synthase large subunit	Valine, leucine and isoleucine biosynthesis
SACE_5748	L-Serine[c] + Indole[c] <=> L-Tryptophan[c] + H2O[c]	tryptophan synthase subunit beta	Glycine, serine and threonine metabolism
SACE_5749	L-Serine[c] + Indole[c] <=> L-Tryptophan[c] + H2O[c]	tryptophan synthase subunit beta	Glycine, serine and threonine metabolism
SACE_5750	2cpr5p[c] <=> Indoleglycp[c] + CO2[c] + H2O[c]	indole-3-glycerol-phosphate synthase	Phenylalanine, tyrosine and tryptophan biosynthesis
SACE_5755	Phosphoribosyl-AMP[c] + H2O[c] => 5-(5-Phospho-D-ribosylaminoformimino)-1-(5-phosphoribosyl)-imidazole-4-carboxamide[c]	phosphoribosyl-AMP cyclohydrolase	Histidine metabolism
SACE_5756	N-(5'-Phospho-D-1'-ribulosylformimino)-5-amino-1-(5''-phospho-D-ribosyl)-4-imidazolecarboxamide[c] + L-Glutamine[c] => eig3p[c] + aicar[c] + L-Glutamate[c]	imidazoleglycerol-phosphate synthase cyclase	Histidine metabolism
SACE_5759	5-(5-Phospho-D-ribosylaminoformimino)-1-(5-phosphoribosyl)-imidazole-4-carboxamide[c] => N-(5'-Phospho-D-1'-ribulosylformimino)-5-	1-(5-phosphoribosylamino)methylideneamino] imidazole-4-carboxamide isomerase	Histidine metabolism

	amino-1-(5''-phospho-D-ribosyl)-4-imidazolecarboxamide[c]		
SACE_5761	N-(5'-Phospho-D-1'-ribulosylformimino)-5-amino-1-(5''-phospho-D-ribosyl)-4-imidazolecarboxamide[c] + L-Glutamine[c] => eig3p[c] + aicar[c] + L-Glutamate[c]	imidazoleglycerol-phosphate synthase cyclase	Histidine metabolism
SACE_5775	ptd1ino160[c] + H2O[c] => i1p[c] + 1,2-Diacyl-sn-glycerol[c] + 2 H[c]	non-hemolytic phospholipase C precursor	Inositol phosphate metabolism
SACE_5776	eig3p[c] => imacp[c] + H2O[c]	imidazoleglycerol-phosphate dehydratase	Histidine metabolism
SACE_5801	Pyridine-2,3-dicarboxylate[c] + prpp[c] => nicrnt[c] + Diphosphate[c] + CO2[c]	nicotinate-nucleotide pyrophosphorylase	Nicotinate and nicotinamide metabolism
SACE_5802	L-Aspartate[c] + O2[c] => Iminoaspartate[c] + H2O2[c]	L-aspartate oxidase	Nicotinate and nicotinamide metabolism
SACE_5803	Iminoaspartate[c] + dhap[c] => Pyridine-2,3-dicarboxylate[c] + 2 H2O[c] + Orthophosphate[c]	quinolinate synthetase [SP:tr:A4FLR2_SACEN]	Nicotinate and nicotinamide metabolism
SACE_5912	(2S,4S)-4-Hydroxy-2,3,4,5-tetrahydrodipicolinate[c] + NADH[c] + H[c] => thdp[c] + NAD[c] + H2O[c]	4-hydroxy-tetrahydrodipicolinate reductase	Lysine biosynthesis
SACE_5918	ATP[c] + Riboflavin[c] => ADP[c] + FMN[c]	riboflavin kinase / FMN adenylyltransferase	Riboflavin metabolism
SACE_5992	2mecdp[c] + 2 fdxrd[c] <=> h2mb4p[c] + H2O[c] + 2 fdxox[c]	4-hydroxy-3-methylbut-2-en-1-yl diphosphate synthase	Terpenoid backbone biosynthesis
SACE_5994	1-Deoxy-xu5p_D[c] + NADPH[c] + H[c] => 2me4p[c] + NADP[c]	1-deoxy-xu5p_D reductoisomerase	Terpenoid backbone biosynthesis
SACE_6034	CTP[c] + Phosphatidate[c] => Diphosphate[c] + CDP-diacylglycerol[c]	phosphatidate cytidylyltransferase	Glycerophospholipid metabolism
SACE_6137	dhap[c] + NADH[c] + H[c] => glyc3p[c] + NAD[c]	glycerol-3-phosphate dehydrogenase (NAD(P)+)	Glycerophospholipid metabolism
SACE_6143	(R)-2-Methylmalate[c] => 2-Methylmaleate[c] + H2O[c]	3-isopropylmalate dehydratase small subunit	Valine, leucine and isoleucine biosynthesis

SACE_6144	(R)-2-Methylmalate[c] => 2-Methylmaleate[c] + H2O[c]	3-isopropylmalate dehydratase small subunit	Valine, leucine and isoleucine biosynthesis
SACE_6157	(S)-2-Aceto-2-hydroxybutanoate[c] <=> (R)-3-Hydroxy-3-methyl-2-oxopentanoate[c]	ketol-acid reductoisomerase	Valine, leucine and isoleucine biosynthesis
SACE_6158	Pyruvate[c] + 2-Oxobutanoate[c] => (S)-2-Aceto-2-hydroxybutanoate[c] + CO2[c]	acetolactate synthase large subunit	Valine, leucine and isoleucine biosynthesis
SACE_6159	Pyruvate[c] + 2-Oxobutanoate[c] => (S)-2-Aceto-2-hydroxybutanoate[c] + CO2[c]	acetolactate synthase large subunit	Valine, leucine and isoleucine biosynthesis
SACE_6270	Phosphoenolpyruvate[c] + uacgam[c] => uaccg[c] + Orthophosphate[c]	UDP-N-acetylglucosamine 1-carboxyvinyltransferase	Amino sugar and nucleotide sugar metabolism
SACE_6435	Phosphoenolpyruvate[c] + skm3p[c] <=> Orthophosphate[c] + 3psme[c]	3-phosphoshikimate 1-carboxyvinyltransferase	Phenylalanine, tyrosine and tryptophan biosynthesis
SACE_6485	5caiz[c] <=> 5ai4izc[c]	5-(carboxyamino)imidazole ribonucleotide mutase	purine metabolism
SACE_6486	ATP[c] + airt[c] + HCO3[c] => ADP[c] + Orthophosphate[c] + 5caiz[c]	5-(carboxyamino)imidazole ribonucleotide synthase	purine metabolism
SACE_6492	hisp[c] + H2O[c] => L-Histidinol[c] + Orthophosphate[c]	L-Histidinol-phosphate phosphohydrolase	Histidine metabolism
SACE_6664	10fthf[c] + aicar[c] <=> Tetrahydrofolate[c] + fprica[c]	bifunctional phosphoribosylaminoimidazolecarboxamide formyltransferase/IMP cyclohydrolase	purine metabolism
SACE_6665	10fthf[c] + 5'-Phosphoribosylglycinamide[c] <=> Tetrahydrofolate[c] + 5'-Phosphoribosyl-N-formylglycinamide[c]	phosphoribosylglycinamide formyltransferase	purine metabolism
SACE_6779	gam6p[c] <=> gam1p_A[c]	putative phosphoglucomutase/phosphomannomutase	Amino sugar and nucleotide sugar metabolism

SACE_6888	hexdp[c] + ipdp[c] => hepdp[c] + Diphosphate[c]	SACE_6888polyprenyl diphosphate synthase component	Terpenoid backbone biosynthesis
SACE_6904	2-Demethylmenaquinone[c] + amet[c] => Menaquinone[c] + ahcys[c]	ubiquinone/menaquinone biosynthesis methyltransferase	Ubiquinone biosynthesis
SACE_6913	Isochorismate[c] + 2-Oxoglutarate[c] => 2sephchc[c] + CO2[c]	2-succinyl-6-hydroxy-2,4-cyclohexadiene-1-carboxylate synthase/2-oxoglutarate decarboxylase	Ubiquinone biosynthesis
SACE_6914	2-Succinylbenzoyl-CoA[c] => 1,4-Dihydroxy-2-naphthoyl-CoA[c] + H2O[c]	naphthoate synthase	Ubiquinone biosynthesis
SACE_6916	ATP[c] + 2-Succinylbenzoate[c] + CoA[c] => AMP[c] + Diphosphate[c] + 2-Succinylbenzoyl-CoA[c]	O-succinylbenzoic acid--CoA ligase	Ubiquinone biosynthesis
SACE_6917	1,4-Dihydroxy-2-naphthoate[c] + octdp[c] => 2-Demethylmenaquinone[c] + Diphosphate[c] + CO2[c]	1,4-dihydroxy-2-naphthoate octaprenyltransferase	Ubiquinone biosynthesis
SACE_6959	1yp5c_S[c] + NADH[c] + H[c] <=> L-Proline[c] + NAD[c]	pyrroline-5-carboxylate reductase	Arginine and proline metabolism
SACE_6978	uaccg[c] + NADH[c] + H[c] => UDP-N-acetylmuramate[c] + NAD[c]	UDP-N-acetylenolpyruvoylglucosamine reductase	Amino sugar and nucleotide sugar metabolism
SACE_7067	Chorismate[c] <=> Prephenate[c]	chorismate mutase	Phenylalanine, tyrosine and tryptophan biosynthesis
SACE_7111	4adcho[c] => 4-Aminobenzoate[c] + Pyruvate[c]	amino acid aminotransferase	Folate biosynthesis
SACE_7124	ATP[c] + fpram[c] => ADP[c] + Orthophosphate[c] + airt[c]	phosphoribosylaminoimidazole synthetase	purine metabolism
SACE_7125	L-Glutamine[c] + prpp[c] + H2O[c] => 5-Phosphoribosylamine[c] + Diphosphate[c] + L-Glutamate[c]	amidophosphoribosyltransferase	Alanine, aspartate and glutamate metabolism
SACE_7150	ATP[c] + 5ai4izc[c] + L-Aspartate[c] <=> ADP[c] + Orthophosphate[c] + 15aics[c]	phosphoribosylaminoimidazole-succinocarboxamide synthase	purine metabolism
SACE_7153	15aics[c] <=> Fumarate[c] + aicar[c]	adenylosuccinate lyase	purine metabolism

SACE_7168	ATP[c] + 5-Phosphoribosylamine[c] + Glycine[c] => ADP[c] + Orthophosphate[c] + 5'-Phosphoribosylglycinamide[c]	phosphoribosylamine--glycine ligase	purine metabolism
SACE_7173	GTP[c] + IMP[c] + L-Aspartate[c] => GDP[c] + Orthophosphate[c] + dcamp[c]	adenylosuccinate synthetase	Alanine, aspartate and glutamate metabolism
